# A Case of Pulmonary Hæmorrhage, with Remarks

**Published:** 1793

**Authors:** William Davidson

**Affiliations:** Apothecary in London.


### XI. *A Caſe of Pulmonary Hæmorrhage, with Remarks. By Mr.* William Davidſon, *Apothecary in London.*

HAVING, in the third volume of Medical Facts and Obſervations, related two caſes of pulmonary hæmorrhage, and from them endeavoured to demonſtrate that the proximate cauſe of active hæmorrhage often conſiſts in diſtention and conſequent increaſed action of the blood veſſels, and to point out that abſtinence from liquids is a principal mean of removing this diſtention; I ſhall now beg leave to add another, which lately occurred, in further confirmation of that doctrine, and of the advantages reſulting from ſuch a method of treatment. The patient was a robuſt man, of a ſanguineous temperament, and about ſixty-four years of age. He had been affected with a ſevere cough for near four months before the preſent attack; and during the laſt ſeven or eight weeks had been ſpitting blood, mixed with a yellow expectoration; but without any pain in or about the cheſt.

 Being

Being ſent for on the fourth day of October, 1792, I found him in an inſenſible ſtate, as if from oppreſſion of the brain, with occaſional ſtrong contractions or convulſions of the right arm. His pulſe was ſtrong, frequent and full; his tongue was furred, and his breathing laborious. He had been juſt brought into the houſe from a Stone-maſon's yard, where he was employed in ſawing.

The perſons about him informed me, that, while at work, he was ſeized with a fit of coughing, and brought up about three or four pints of blood; that he ſoon after became inſenſible, and was immediately brought home. Conſidering the great hæmorrhage which had taken place, and the apoplectic ſymptoms now preſent, I conceived my patient to be in conſiderable danger, and that the moſt active method of relieving him ſhould be adopted: accordingly ſixteen ounces of blood were inſtantly taken from a large orifice in the arm. The blood, when coagulated, was covered with the buff coat, as it is called. A few minutes after the operation he became ſenſible, and complained of great pain in the anterior part of the cheſt; which, he ſaid, he had firſt perceived that morning.

About

About half an hour after the bleeding, he took a purging draught, chiefly compofed of magnefia vitriolata. A large blifter was alfo applied to the breaft. He was particularly directed to refrain from drink during the operation of the purgative medicine; and, if thirfty, only to moiften his mouth and throat with a little barley water.

In the evening I found him fenfible, with lefs fever, his cough quiet, his breaft eafier, and he had not brought up much blood. His medicine had purged him feveral times.

A faline draught containing twenty drops of antimonial wine was now directed to be taken every fix hours; he was ftrictly enjoined to drink about half a pint only of liquid during the firft twenty-four hours; and in every other refpect to adhere rigidly to the antiphlogiftic regimen.

October 5th, he had refted pretty well, and expectorated about an ounce or two only of blood, which was chiefly in coagula: his breaft was eafier, but ftill a little tight; his pulfe was much improved, and his fkin was cool and moift. He had little thirft, and his tongue was lefs furred. The ufe of the faline draught was continued, and the opening draught was directed to be repeated in the morning. Being so much better he was now allowed a pint of liquid (including tea, &c.) in the twenty-four hours, and the same quantity only was permitted every day during the whole of his illness.

October 6th, he was still much better: he had rested well, had less cough, less fever, little bloody expectoration, and his pulse was nearly natural: his chest was much easier.

From this time to the 12th he continued gradually to recover. He had no expectoration of blood after the 8th, but the saline draught, and likewise the purgative medicine, were occasionally repeated, and he persevered in the limited use of liquids till the 12th, when I thought it unnecessary to visit him any longer. His pulse was then sixty-eight in a minute, and he was apparently in good health, only a little weak.

I afterwards learned that, contrary to my directions, he went upon duty, as patrole, on the Monday following, the 15th day of October.

Considering, therefore, the nature of this office, the season of the year, the age of the patient, and the short time since his recovery, it it cannot ſeem ſurpriſing that the diſeaſe was reproduced; accordingly, on the 25th, he was again ſeized with fever, difficult breathing, cough, and hæmorrhage. He continued, notwithſtanding this return of the complaint, to attend his duty regularly until Sunday the 28th, when he was again taken with conſiderable bleeding, while on the patrole, and inſtantly expired.

On Tueſday the 30th, having an opportunity of inſpecting the body, the following appearances preſented themſelves:—the thorax and abdomen being laid open, we obſerved on the anterior ſurface of the right lung an incipient inflammation, which, however, could not account for the patient's death, for, on further examination, it ſeemed evidently to be occaſioned by the hæmorrhage. There were alſo ſome adheſions, apparently rather veſtiges of former than of any recent inflammation. There were no tubercles. A portion of the aorta was oſſified. All the abdominal viſcera were ſound. In the ſtomach there was ſome coagulated blood, which had been ſwallowed; but there was not the ſmalleſt eroſion of its coats.

In the two former cafes of hæmoptyfis, I have noticed the great difficulty of curing a ruptured veffel in the lungs, on account of their conftant motion, and the great quantity of blood circulated through them; but that this difficulty might be, in general, overcome by a fteady adherence to the plan of cure there recommended, viz. moderate bleeding and purging, but particularly abftinence from liquids. The fuccefs attending the treatment of the prefent cafe muft evidently eftablifh the fuperiority of that method of cure over every other hitherto recommended. Here a blood veffel, of confiderable magnitude, was ruptured in a part of the body which, from its natural office, muft be in perpetual action, and where no local application could be made; yet this rupture was healed in almoft as fhort a time as the moft experienced Surgeon can heal an external accident of the fame nature, even with the affiftance of compreffes and bandages. For example, I have feen a rupture of fome fuperficial veffels require thefe applications for many days. It may be faid that the bleeding, which has been more or lefs plentifully ufed (I mean as to quantity, for it was never ufed more than once in each cafe) according to the urgency of the the ſymptoms, was the chief mean of cure. But a practitioner, who has ſeen a patient blooded twelve or thirteen times for an hæmorrhage from the lungs and ſtill ſink under the diſeaſe, will not readily ſubſcribe to this opinion.

It may be neceſſary to obſerve that the patient to whom I allude was allowed to take, and actually did drink, ſeveral quarts of diluents in the twenty-four hours. But ſuppoſing he had recovered; after ſuch loſs of blood he muſt have remained infirm for many months: whereas this patient, who was ſo ſoon relieved by abſtinence from liquids, had he been in eaſy circumſtances, and could he have kept from labour and improper expoſure to the night air, for another week or two, might have obtained a perfect and permanent cure, without any particular diminution of bodily ſtrength.

Reſpecting the other medicines, they were doubtleſs of ſervice, and conſpired to effect a cure, which, had the uſual quantity of diluents been uſed, I am convinced, would, notwithſtanding, have been much more tedious. For in vain do practitioners attempt to leſſen diſtention by emptying the veſſels, either by purging or bleeding, if they are immediately filled again by plentiful drinking. The ſpare uſe of liquids, therefore, may juſtly be conſidered as one of the greateſt improvements in the modern treatment of hæmorrhage: and particularly in hæmorrhages from the lungs. And why ſhould not the idea be carried farther? Indeed, from ſome caſes I have lately attended, I think I may venture to aſſert, that, in all diſeaſes of the lungs, moderate drinking will be of ſervice. For ſeeing they are a congeries of veſſels, if theſe veſſels are overfilled, or kept in a continued ſtate of diſtention, they may ſo preſs upon one another that their healthy actions ſhall be either prevented or greatly impeded, particularly the actions of the abſorbent ſyſtem: whereas, if they are but moderately filled, the different ſyſtems of veſſels are left more at liberty to exerciſe their reſpective functions, either in the buſineſs of health, or in the removal of diſeaſe. When tubercles are formed in the lungs, why ſhould they not be abſorbed? We know that the moſt ſolid tumors in other parts of the body frequently diſappear; and that even bone itſelf is capable of being abſorbed, as is clearly demonſtrated by the different changes which take place in it as well in health as in diſeaſe. And in the lungs there are many abſorbent veſſels, which, if their actions tions were not leſſened or prevented, might ſoon remove the moſt confirmed induration of their ſubſtance. As emetics are powerful promoters of abſorption, is it not on this principle that many patients, ſeemingly labouring under tubercles of the lungs, have been cured by vomits, particularly of the ſtronger kind? I hope the time is not far diſtant when practitioners, being better acquainted with the laws and functions of this important ſyſtem, ſhall be enabled to direct its actions with more certainty, either in removing a tubercle or the moſt ſchirrhous tumor. But when this happy period arrives it can only be carried into effect by a proper regulation of the quantity of liquids; and, in general, a diminution of the uſual preſcribed quantities. Perhaps the advantages ariſing to conſumptive patients from a warm climate and the uſe of flannel, are principally from their doing the ſame thing as abſtinence from liquids, viz. determining the tide of circulation to the ſurface of the body, and thus leaving the veſſels of the lungs more empty, and, therefore, more ready to recover themſelves when under the influence of diſeaſe.

*Queen Anne Street Eaſt,*

*January* 14*th*, 1793.

